# Magnetic Induction Tomography Spectroscopy for Structural and Functional Characterization in Metallic Materials

**DOI:** 10.3390/ma13112639

**Published:** 2020-06-09

**Authors:** Imamul Muttakin, Manuchehr Soleimani

**Affiliations:** Engineering Tomography Laboratory (ETL), Department of Electronic and Electrical Engineering, University of Bath, Claverton Down, Bath BA2 7AY, UK; I.Muttakin@bath.ac.uk

**Keywords:** spectral MIT, metal characterization, Cole–Cole plot

## Abstract

Magnetic induction tomography (MIT) is a powerful imaging system for monitoring the state of metallic materials. Tomographic methods enable automatic inspection of metallic samples making use of multi-sensor measurements and data processing of eddy current-based sensing from mutual inductances. This paper investigates a multi-frequency MIT using both amplitude and phase data. The image reconstruction algorithm is based on a novel spectrally-correlative total variation method allowing an efficient and all-in-one spectral reconstruction. Additionally, the paper shows the rate of change in spectral images with respect to the excitation frequencies. Using both spectral maps and their spectral derivative maps, one can derive key structural and functional information regarding the material under test. This includes their type, size, number, existence of voids and cracks. Spectral maps can also give functional information, such as mechanical strains and their thermal conditions and composition.

## 1. Introduction

Material characterization as well as substance examination are important procedures in many sectors. A comprehensive knowledge on a sample is desired before, during, and after a process. In the case of a metallic target, passive electromagnetic properties such as conductivity and permeability convey crucial information about its structural and functional traits. This leads to the employment of inspection techniques based on electromagnetic measurements.

Eddy currents are widely used for examining a metal embodiment. Their application for non-destructive testing has been continuously developed and adapted [1]. In addition to a direct defect observation, electrical conductivity is also measured using the eddy current method [2,3]. Commonly, a coil probe is employed to detect magnetic fields, primarily from an exciter and secondarily from the target’s response, then both amplitude and phase in the induced signal are extracted to give an indication of the target’s characteristics. The coil probe is relatively more sensitive to drastic disruptions in the eddy current flow with high dynamic range.

The multi-frequency technique expands the capability of the single-frequency technique. The wide-band signal can profile the structural depth inside metallic materials [4]. Therefore, it is able to accurately examine the properties since the use of only a single lower frequency results in a reduced signal-to-noise ratio in the detection. Furthermore, data at different frequencies can be correlated to characterize the object under test. Some reports studied the spectral response of pulsed eddy current [5], a multi-frequency technique for material characterization [6], and frequency sweep and impedance normalization methods [7].

The measurement of field and impedance variations also facilitate conductivity imaging in materials. The inversion of inductive spectra was employed to determine characteristics such as magnetic permeability, electrical conductivity and thickness [8]. Multiple frequency data can be reconstructed simultaneously to exploit the correlation among conductivity distributions at different frequencies [9]. Thus, different excitation frequencies enrich the information, improving the inverse method, and strengthens the system against experimental noises [10].

Magnetic induction spectroscopy (MIS) is a method for measuring the conductivity spectrum using a non-destructive and contactless technique [11]. The term was introduced in [12] which then has been followed by subsequent works to measure the conductivity spectrum using gradiometer coil sensors [13], utilizing differential methods [14] and signal improvement schemes [15]. The use of inductance spectroscopy has been exploited for imaging both continuous conductivity profiles [16] and the permeability distribution of a layered sample [17].

Applications of magnetic induction spectroscopy have been found in biological and industrial areas. The design of a practical MIS system was reported specifically for bioelectrical impedance spectroscopy on yeast suspension in saline, fruits and tissue [18]. Recent spectroscopic bioimpedance measurements were described for industrial-scale agricultural produce [19]. In the medical domain, gradiometer sensors were designed to perform in vivo spectroscopic measurements on a human hand [20]; meanwhile electromagnetic phase-shift spectroscopy was developed to diagnose brain oedema and brain hematoma [21]. On the other hand, industrial implementations vary from detection, classification and characterization. Spectroscopic metal detection provides rich and distinctive information about a target to help reduce the false alarm rate in landmine detection [22], as well as for buried pipeline tracing in difficult terrain [23]. Additionally, the metal recycling sector requires sorting processes, for which a classification of non-ferrous metals based on MIS was developed in [24]. A particular frequency feature was proposed for imaging a welding cross-section [25]. Internal material structure examinations using inductance spectroscopy measurements were presented in many investigations, such as in [26,27,28]. The demand for in-line monitoring of phase transformation in the steel industry is answered by multi-frequency electromagnetic instruments [29,30,31,32].

Our previous studies on MIS deal with hardware development [33], as well as characterizing ferromagnetic materials [34]. Various image reconstruction methods for soft field tomography techniques have been reviewed in [35]. The Tikhonov regularization method is commonly used in solving the MIT inverse problem. This least-square solution has disadvantages, such as an overly smoothed image so that boundaries between samples become obscure. The use of an L1-norm regularization, e.g., total variation (TV), can improve the MIT image quality. It is a more suitable method for both sharp edges and high contrast. However, this method faces difficulty in low-contrast recovery. The iterative technique has been proposed to fix the low-contrast recovery problem [36]. It provides a sequence of solutions which allows recovering the contrast lost. In this work, we aim to inspect metallic samples with functional and structural variations. The algorithm is proposed for spectrally correlative imaging as magnetic induction tomography spectroscopy (MITS). Spatial maps of the conductive spectrum and its derivative are presented.

## 2. Experimental Setup

**Figure 1:**
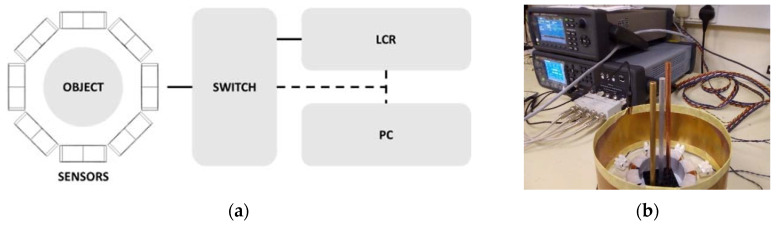
Measurement setup: (**a**) sketch of the sensor array and object in the sensing region, switch, LCR meter and PC connection (signal, solid-line; control, dashed-line); (**b**) photograph of the system.

A multi-frequency data collection is proposed to cover a wide range of the frequency spectrum. The experimental setup is depicted in Figure 1.

The sensors comprise eight coils arranged encircling the sensing space with diameter of 50 mm. An individual sensor is an off-the-shelf induction coil with a self-inductance value of 0.1 mH and 0.3 Ω intrinsic resistance. Amplitude and phase measurements of mutual impedance between sensors (transmitting-receiving coils) are acquired by an LCR meter (frequency range 20 Hz–300 kHz). For eight-coil arrays, the switch module is introduced to accommodate 28 independent coil-pair measurements. Synchronous operation between mutual coil selection (switch) and current-source with potential-sense (LCR) is controlled by the instrument programming interface on PC. It takes approximately 10 min to collect a complete cycle of measurement.

**Figure 2:**
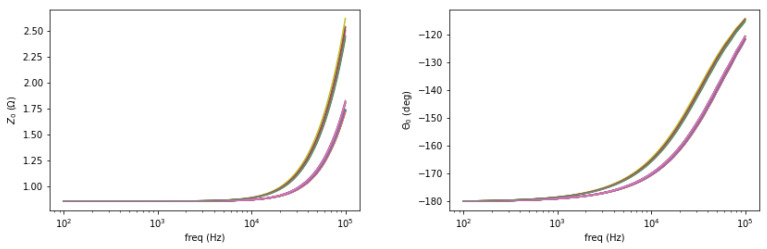
Free space background measurement of amplitude (**left**) and phase (**right**) for 28 coil-pair combinations.

**Figure 3:**
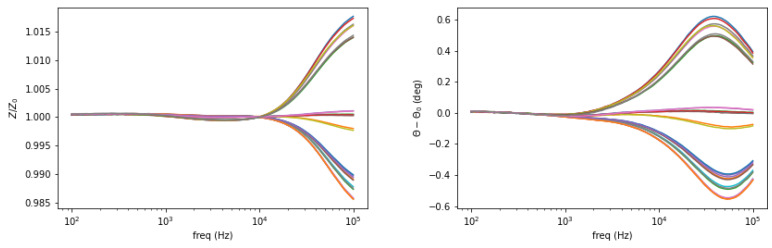
Amplitude (**left**) and phase (**right**) spectrum of a test sample plotted against the background.

Background measurement is taken for free space (air) condition in the sensing region as a reference. The amplitude spectrum is formed as the normalized mutual impedance of an object (Z) against free space (Z_0_), whereas the phase spectrum is the difference between the measured phase in the presence of an object (θ) and that of free space (θ_0_), shown in Figure 2 and Figure 3.

For simplicity, norm values of 28 measurements are presented to show spectral plots of the upcoming investigated cases. Fundamental plots are given for several cases: conductivity, size and structure variations.

**Figure 4:**
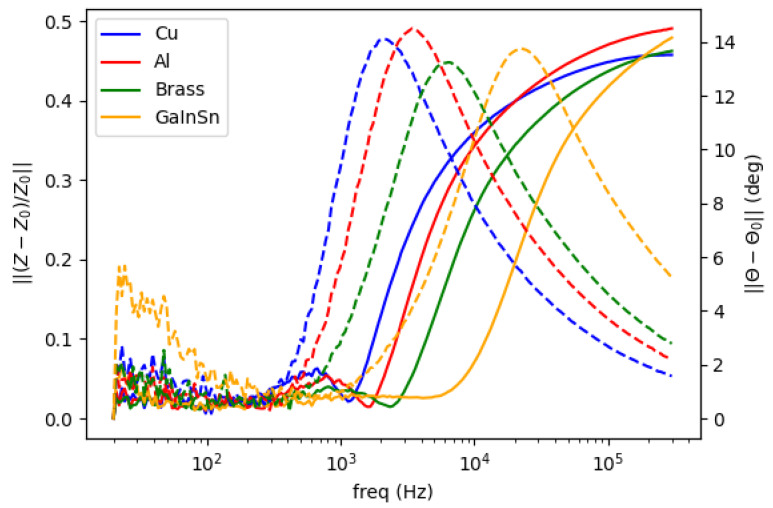
Spectral plot for metallic samples (conductivity variations). Solid line for amplitude ratio (left vertical axis); dashed line for phase difference (right vertical axis).

Figure 4 shows spectral plot for different metallic samples whose conductivity are varied. From the highest to the lowest are copper (58.4 MS/m), aluminum (26.3 MS/m), brass (16.1 MS/m) and Galinstan (3.2 MS/m). The left vertical axis is the normalized amplitude for the solid-line plot, while the phase difference for the dashed-line plot is on the right axis. The inclinations (for amplitude) and peaks (for phase) are distributed following the respective conductivity values.

**Figure 5:**
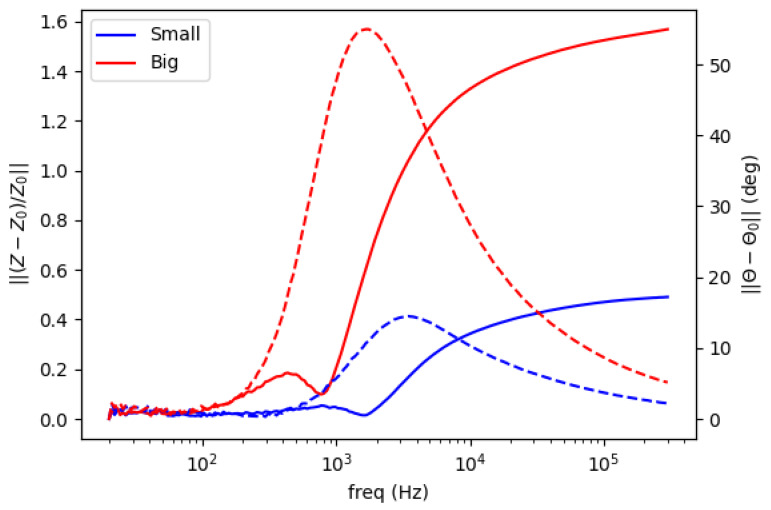
Spectral plot for size variations. Solid line for amplitude ratio (left vertical axis); dashed line for phase difference (right vertical axis).

Figure 5 shows spectral plot of aluminum rods with different diameter (small = 0.25 inch, large = 0.5 inch). Both amplitude and phase curves have larger scales for a larger object’s size.

**Figure 6:**
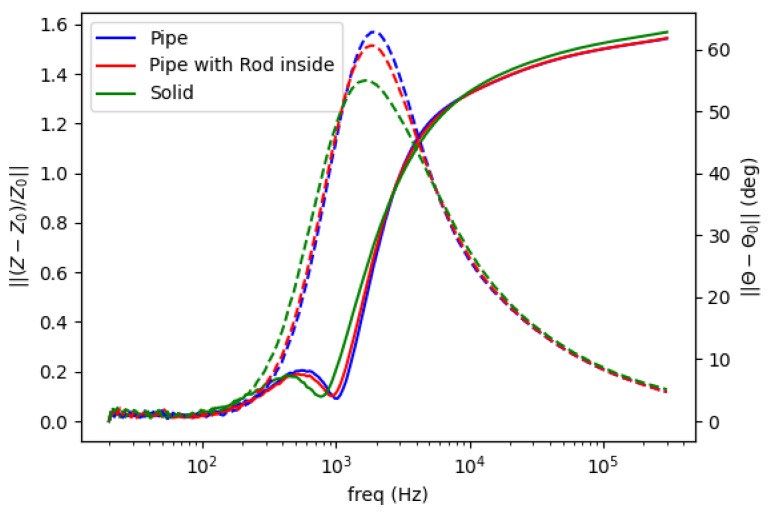
Spectral plot for metallic structure. Solid line for amplitude ratio (left vertical axis); dashed line for phase difference (right vertical axis).

Figure 6 shows spectral plot of aluminum samples with different structures: pipe (hollow cylinder with outer diameter 0.5 inch and inner diameter 0.4 inch), pipe (as previous) with 0.25 inch aluminum rod inside, and solid aluminum rod 0.5 inch. There are no significant differences in amplitude curves, whereas in phase curves, peaks’ signatures reveal distinct fashions according to the object’ structures.

**Figure 7:**
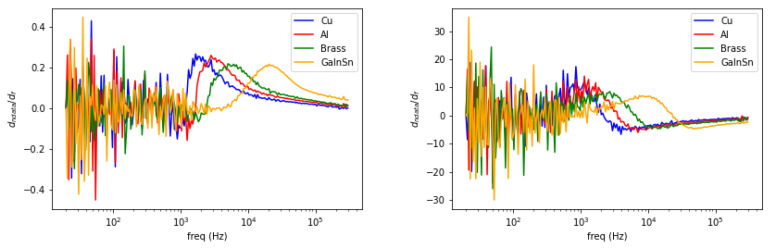
Spectral derivative for metallic samples with conductivity variations (**left**: amplitude, **right**: phase).

**Figure 8:**
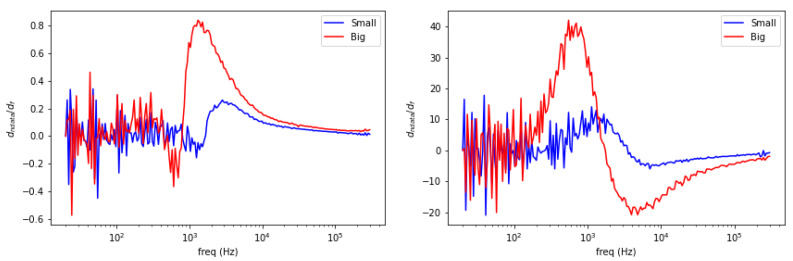
Spectral derivative for size variations (**left**: amplitude, **right**: phase).

**Figure 9:**
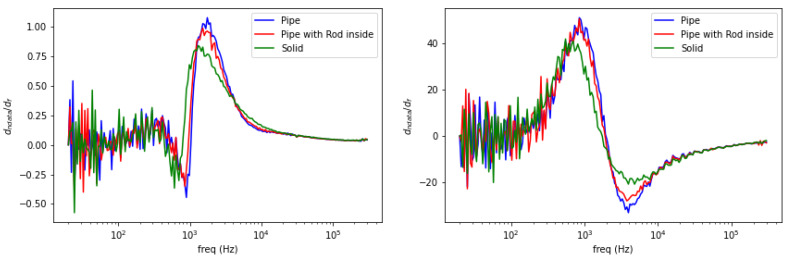
Spectral derivative for metallic structures (**left**: amplitude, **right**: phase).

Observing the aforementioned cases, spectral characteristics can be further treated into frequency derivative to classify metallic materials. These are shown in the following Figure 7, Figure 8 and Figure 9.

Spectral data measured from coil arrays are then transformed into imaging domain utilizing reconstruction technique in magnetic induction tomography. The 2D spatial position is evaluated along the diameter across the centre (see Figure 1). Therefore, it turns into 1D values of image versus frequencies.

## 3. Sensor Modelling

MIT utilizes an array of inductive coils, distributed equally around an imaging region, to visualize the electromagnetic property distribution of the electrical conductivity of an imaging subject. The imaging principle is based on the laws of induction and eddy currents which are induced in an AC magnetic field [37]. The formulation can be obtained from the Maxwell’s equations [38,39]:

(1) $$\nabla \times \frac{1}{\mu }\nabla \times A+j\omega \sigma A=J_{s}$$

where ω is the angular frequency, μ is the permeability, A is the total magnetic vector potential as a result of the effect of eddy current induced by the electrical conductivity σ and the current source $J_{s}$.

Equation (1) can be solved by approximating the system as a combination of linear equations in small elements with appropriate boundary conditions using the Galerkin’s approximation [40]:

(2) $$\int_{\Omega_{c}+\Omega_{s}}\left(\nabla \times N_{i}\cdot \frac{1}{\mu }\nabla \times A\right)dv+\int_{\Omega_{c}+\Omega_{s}}(j\omega \sigma N_{i}\cdot A)dv=\int_{\Omega_{s}}\left(\nabla \times N_{i}\cdot T_{s}\right)dv$$

where $T_{s}$ is the electric vector potential and $J_{s}=\nabla \times T_{s}$, $N_{i}$ is the linear combination of edge shape functions, and $\Omega_{C}$ and $\Omega_{S}$ are the eddy current region and current source region or excitation coil region, respectively.

The right-hand side of Equation (2) can be solved with the aid of Biot–Savart Law. When $J_{0}$ is the unit current density passing through coil, the measured induced voltage in sensing coil can be calculated:

(3) $$V_{mn}=-j\omega \int_{\Omega_{s}}\left(A\cdot J_{0}\right)dv$$

Then Jacobian matrix can be formulated by:

(4) $$J=\frac{\partial V_{mn}}{\partial \sigma_{x}}=-\omega^{2}\frac{\int_{\Omega_{x}}A_{m}\cdot A_{n}dv}{I}$$

where $\sigma_{x}$ is the conductivity of pixel x and $\Omega_{x}$ is the volume of the perturbation, $A_{n}$ is the forward solver of sensor coil excited by unit current, $A_{m}$ is the forward solver of excitation coil m excited with I. Amplitude and phase Jacobian are given:

(5) $$J_{amp}=\frac{V_{r}J_{r}+V_{i}J_{i}}{\left|V\right|}$$

(6) $$J_{phs}=\frac{V_{r}J_{i}-V_{i}J_{r}}{{\left|V\right|}^{2}}$$

where $V_{r}$ and $V_{i}$ are real and imaginary part of the measurement voltage. If we reconstruct real and imaginary part of the impedance, then real and imaginary part of J in Equation (4) can be used. For forward modelling, we used non-ferrous materials, which means relative permeability of 1, and conductivity according to the metal sample.

## 4. Spatio-Spectral Image Reconstruction Algorithm

The linear inverse problem in MITS can be defined as the recovery of a change in complex conductivity Δσ from a change in measured data Δu, where Δu=JΔσ. The Jacobian J is computed by the Fréchet derivative of u with respect to σ [41]. The complex conductivity includes the resistive component and reactive component of the admittivity of the samples under test. In this case, a reference boundary voltage $u_{0}$ is available, where$\;u_{0}=F\left(\sigma_{0}\right)$,$\;\Delta u=u-u_{0}$, and $\Delta \sigma =\sigma -\sigma_{0}.$ Measured data in this case is real and imaginary part of mutual inductance, or amplitude and phase of the mutual inductance.

In spectral imaging, the unknown conductivity changes and data are multidimensional. Let us redefine $\Delta \sigma =\left[{\Delta \sigma }_{1},\ldots ,{\Delta \sigma }_{I}\right]$ and data$\;\Delta u=\left[{\Delta u}_{1},\ldots ,{\Delta u}_{I}\right]$, where$\;\Delta u=\;\tilde{J}\Delta \sigma$, for i=1,…,I, and *I* is the number of spectral frames. It is common to recover each frame independently, but this is not optimal, as it does not exploit redundant information across frames. In this case, previous works have defined the inverse problem as follows [42]:

(7) $$arg\underset{{\Delta \sigma }_{i}}{\min}\phi \left({\Delta \sigma }_{i}\right)\;\;\;s.t.\;\;\;\;\left\|{J\Delta \sigma }_{i}-{\Delta u}_{i}\right\|{}_{2}^{2}\leq \delta ,\;\forall \;i=1,\ldots ,I$$

where $\phi \left({\Delta \sigma }_{i}\right)$ is a convex regularization functional that carries a priori information of the unknown conductivity distribution for a single frame.

This paper proposes a spatio-spectral reconstruction framework that exploits regularization [43]. Spatio-spectral total variation is implemented as MIT images can be well approximated by a piecewise constant function and consecutives frames are expected to be similar. This allows to exploit redundant information across consecutive frames. The spatio-spectral total variation problem can be written as follows [44,45]:

(8) $$arg\underset{\Delta \sigma }{\min}{\left\|\nabla_{x,y,z}\Delta \sigma \right\|}_{1}+{\left\|\nabla_{f}\Delta \sigma \right\|}_{1}\;\;\;s.t.\;\;\;\;\;{\;\|\tilde{J}\Delta \sigma -\Delta u\|}_{2}^{2}\leq \delta$$

where first and second terms correspond to isotropic spatial TV and spectral TV functional, respectively, and where Δσ represents a spectrally correlated conductivity distribution and $\tilde{J}$ is an augmented Jacobian operating on a frame-by-frame basis.

The constrained optimization problem (Equation (7)) can be solved using the split Bregman formulation, which efficiently handled constrained optimization and L1-regularization [46,47]. Using the Bregman iteration, the constrained problem (Equation (7)) is converted to an iterative scheme:

(9) $${\Delta \sigma }^{k+1}=arg\underset{\Delta \sigma }{\min}{\left\|\nabla_{x,y,z}\Delta \sigma \right\|}_{1}+{\left\|\nabla_{f}\Delta \sigma \right\|}_{1}+\overset{I}{\underset{i=1}{\sum }}\frac{\mu }{2}\;\|\tilde{J}\Delta \sigma -{\Delta u}^{k}\|{}_{2}^{2}$$

(10) $${\Delta u}^{k+1}={\Delta u}^{k}-{\;\tilde{J}\Delta \sigma }^{k+1}+\Delta u,$$

where Equation (8) is an unconstrained optimization problem and Equation (9) is a Bregman iteration that imposes the constraint iteratively. The cost function in Equation (8) is still difficult to minimize given the non-differentiability of the TV functional, but this can be easily done with a splitting technique. Including auxiliary variables allow splitting L1- and L2-functional in such a way that they can be solved in separate steps in an easy manner. Images Δσ are given analytically by solving a linear system and L1-functional are solved using shrinkage formulae. To perform the split, we include ${\;d}_{x}=\nabla_{x},{\;d}_{y}=\nabla_{y,\;}{\;d}_{z}=\nabla_{z},{\;d}_{f}=\nabla_{f}$, so Equation (8) becomes:

(11) $$\begin{array}{c} \left({\Delta \sigma }^{k+1},d_{x},d_{y},d_{z},d_{f}\right)=arg\underset{\Delta \sigma ,d_{x},d_{y,}d_{z},d_{f}}{\min}{\left\|\left(d_{x},d_{y},d_{z}\right)\right\|}_{1}+{\left\|d_{f}\right\|}_{1}+\frac{\mu }{2}{\left\|\;\tilde{J}\Delta \sigma -{\Delta u}^{k}\right\|}_{2}^{2}\\ \mathrm{st}.{\;d}_{i}=\nabla_{i}\Delta \sigma , \end{array}$$

Constraints in Equation (10) can be handled using the Bregman iteration as above, which leads to the following iterative scheme:

(12) $$\left(\mu {\tilde{J}}^{T}\tilde{J}+\lambda \underset{i=x,y,z,f}{\sum }\nabla_{i}^{T}\nabla_{i}\right)\Delta \sigma^{k+1}=\mu {\tilde{J}}^{T}\Delta u^{k}+\lambda \underset{i=x,y,z,f}{\sum }\nabla_{i}^{T}\left(b_{i}^{k}-d_{i}^{k}\right)$$

(13) $$d_{i}^{k+1}=\max\left(p^{k}-\frac{1}{\lambda },0\right)\frac{\nabla_{i}\Delta \sigma^{k+1}+b_{i}^{k}}{p^{k}},\;\mathrm{for}\;i=x,y,z$$

(14) $$p^{k}=\sqrt{\underset{i=x,y,z}{\sum }{\left|\nabla_{i}\Delta \sigma^{k+1}+b_{i}^{k}\right|}^{2}}$$

(15) $$d_{f}^{k+1}=\max\left(\left|\nabla_{f}\Delta \sigma^{k+1}+b_{f}^{k}\right|-\frac{1}{\lambda },0\right)\frac{\nabla_{f}\Delta \sigma^{k+1}+b_{f}^{k}}{\left|\nabla_{f}\Delta \sigma^{k+1}+b_{f}^{k}\right|}$$

(16) $$b_{i}^{k+1}=b_{i}^{k}+\nabla_{i}\Delta \sigma^{k+1}-d_{i}^{k+1},\;\mathrm{for}\;i=x,y,z,f$$

(17) $$\Delta u^{k+1}=\Delta u^{k}+\Delta u-\tilde{J}\Delta \sigma^{k+1}$$

Equation (11) is a linear system that can be solved efficiently using a Krylov solver [44,45,48], such as the bi-conjugate gradient stabilized method, which involves only matrix-vector multiplications. Number of Bregman iteration and other imaging parameters are selected empirically.

**Figure 10:**
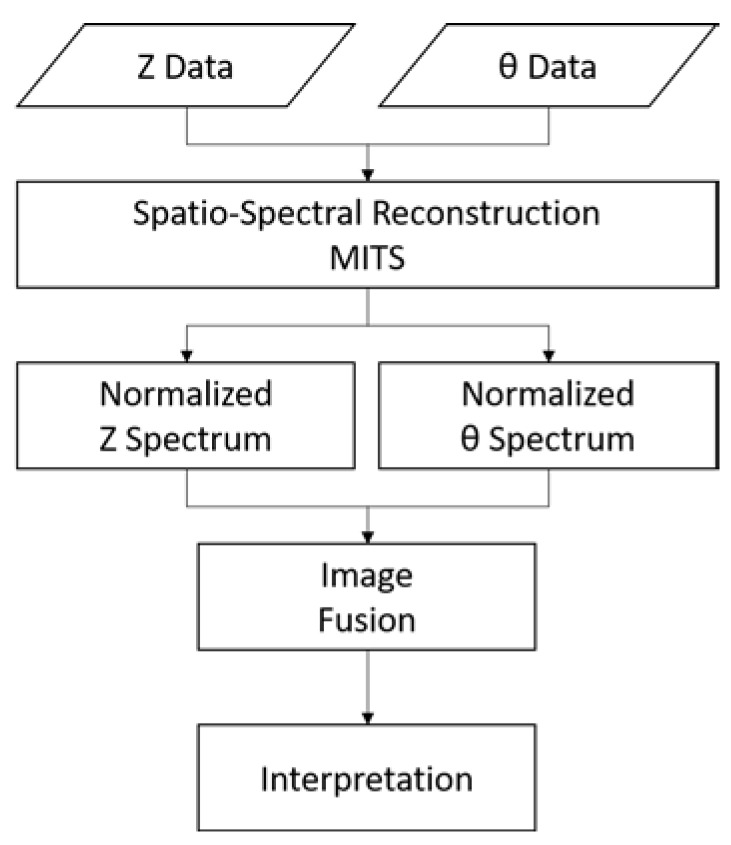
Flowchart of image fusion procedure from amplitude and phase reconstructions.

Distinct information is extracted from the data, where (for example) amplitude gives conductivity level; phase reveals structural detail. Therefore, both data are used and can complement each other. Method to combine amplitude and phase data and/or image is necessary. An example of image fusion procedure is illustrated in Figure 10.

## 5. Results and Analysis

Images were reconstructed for different circumstances of metallic materials: a single conductive sample, different samples at different positions, and non-conductive inclusion in a conductive body. For each condition, a contrast is observed in both measurement data and its spectral derivative. Spatial reconstruction along the frequency is also given. Due to either measurement setup or ambient disturbance, the noise will be present in the data (especially at low frequency). Therefore, additional smoothing is applied.

### 5.1. Single Metal Sample

**Figure 11:**
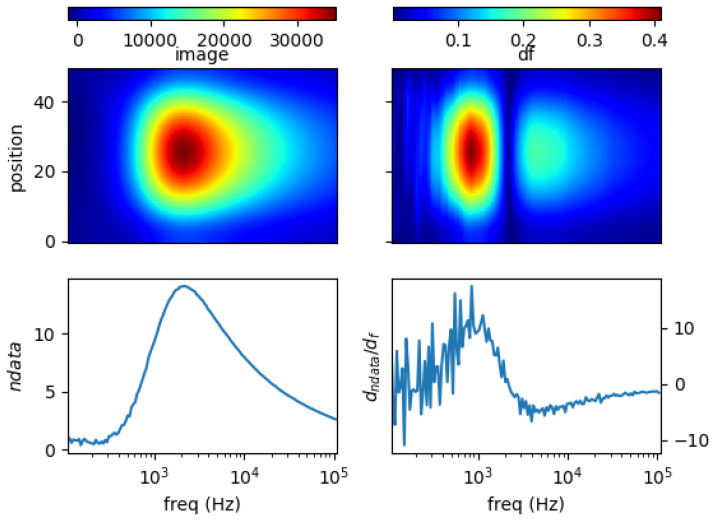
Spectral profile and its derivative (data and reconstructed image values) for a 0.25 inch copper rod (σ = 58.4 MS/m).

**Figure 12:**
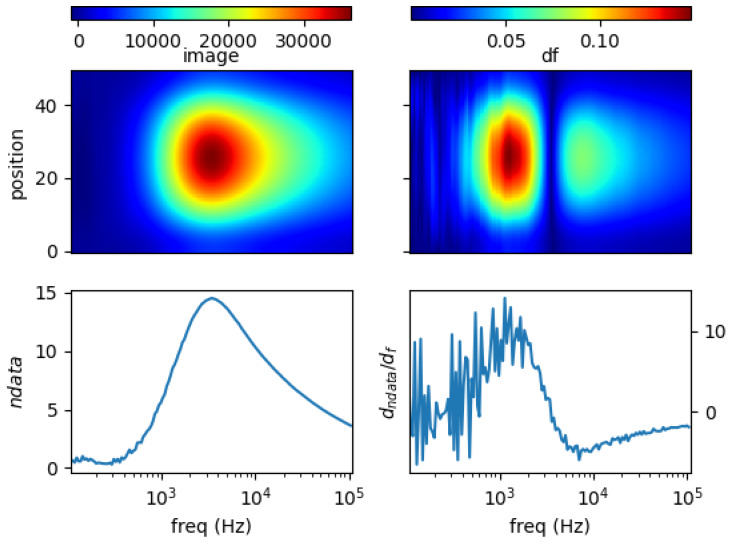
Spectral profile and its derivative (data and reconstructed image values) for a 0.25 inch aluminum rod (σ = 26.3 MS/m).

**Figure 13:**
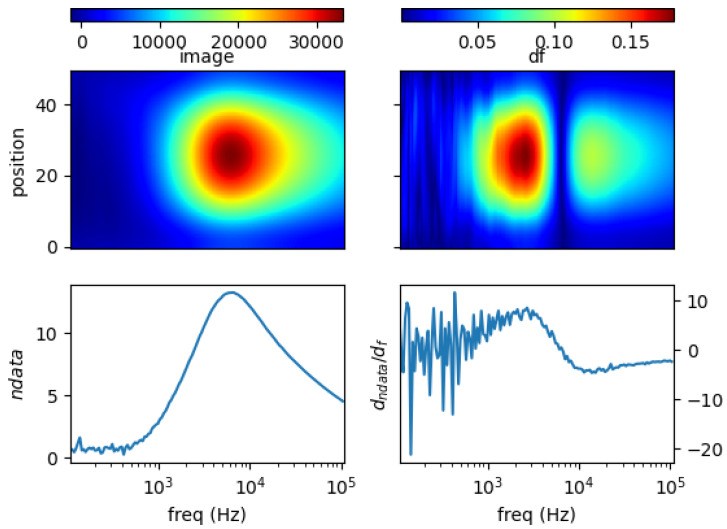
Spectral profile and its derivative (data and reconstructed image values) for brass (σ = 16.1 MS/m) rod 0.25 inch.

**Figure 14:**
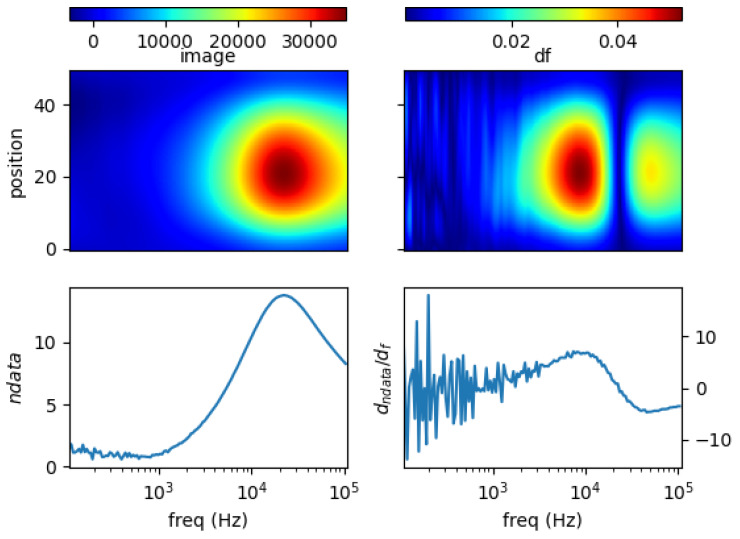
Spectral profile and its derivative (data and reconstructed image values) for liquid GaInSn (σ = 3.2 MS/m) in a 0.25 inch tube.

Figure 11, Figure 12, Figure 13 and Figure 14 show the spectral profiles and the derivative of spectral profile with respect to the frequency for various metal samples. All samples are of the same size and located at the centre of the imaging area. These are the images using phase data from mutual inductance. The maxim phase profile coincides with when the derivative profile goes down to 0. The frequency for which the phase profile is its maximum and the derivative tends to 0 is an indicator of the electrical conductivity of the test sample.

Norm of measurement data (*ndata*) and spectral derivative (*dndata/df*), with the respective spatial reconstruction from data (*image*) and spectral derivative (*df*) are given. For reconstruction figures (top), vertical axis is spatial cross-section (pixelated) where position = 0 is the location of sensor coil 1; and position = 50 is that of sensor coil 5. The horizontal axis represents frequency points associated with the horizontal *log(freq)* axis of the bottom figures.

It can be seen for different samples with different conductivity levels that there are value shifts in phase spectra. The signatures’ locations are marked in spectral derivative data/images, where zero values indicate the extremes of the norm data or the respective reconstructed images.

### 5.2. Different Samples at Different Locations

**Figure 15:**
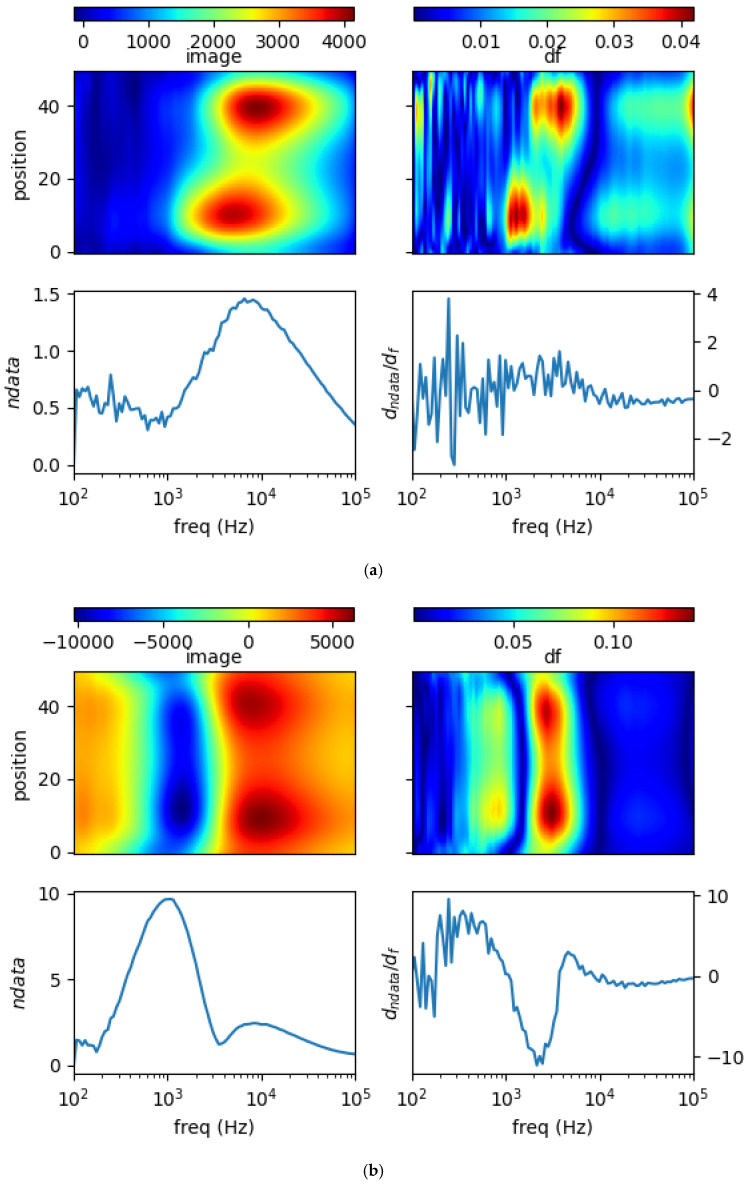
Spectral profile and its derivative (data and reconstructed image values) for (**a**) copper (σ = 58.4 MS/m) rod 0.25 inch at pos = 10 and brass (σ = 16.1 MS/m) rod 0.25 inch at pos = 40; (**b**) brass (σ = 16.1 MS/m) rod 0.25 inch at pos = 10, aluminum (σ = 26.3 MS/m) rod 0.25 inch at pos = 25, and copper (σ = 58.4 MS/m) rod 0.25 inch at pos = 40.

The distribution of different samples at different locations is evaluated. Figure 15a shows a more conductive sample (copper) at position = 10 and less conductive sample (brass) at position = 40. In the reconstructed image, there are two distinct regions depicting the objects, one starts at lower frequency (for higher conductive), whereas the other (for lower conductive) lies at higher frequency. In the spectral derivative there is pronounce line cutting-through ‘position’ that shows the location of both samples, initiating from a more conductive sample at bottom position, then it turns to a less conductive sample at top position.

Figure 15b shows three samples (two at both edges, one at centre) the lowest conductive sample (brass) at position = 10, moderate conductive sample (aluminum) at position = 25, and the highest conductive sample (copper) at position = 40. In the reconstructed image, there should be three different regions representing the objects. However, due to non-uniformity in the sensitivity (highest at near sensors, lower at central region) and further affected by regularisation, the object in the middle is obscure. Still there is an inclined line in the spectral derivative that shows the gradation from low conductive (bottom position), mid-range (centre) to high conductive (top position).

### 5.3. Non-Conductive Inclusion in Conductive Liquid

Particular case likely to be found in the pratical applications is the presence of non-conductive substance in a conductive body.

**Figure 16:**
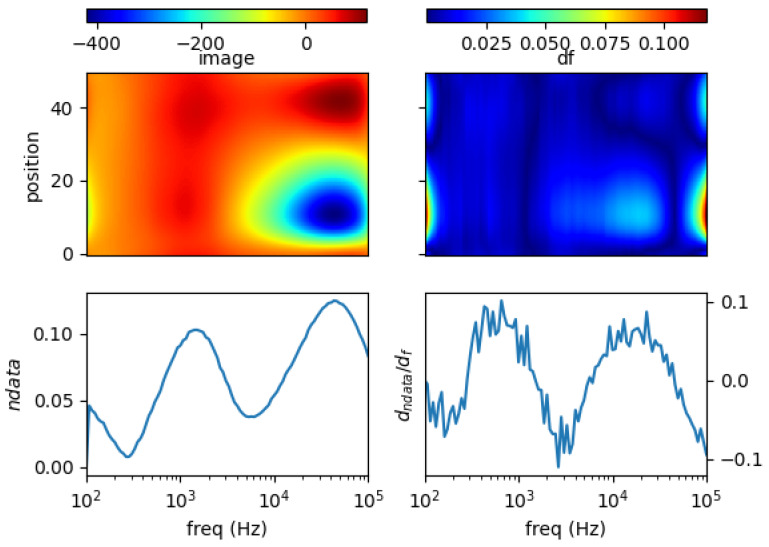
Spectral profile and its derivative (data and reconstructed image values) for wood cube 1 cm^3^ at pos = 10 in liquid GaInSn 35 mL 1 inch tube.

An eutectic GaInSn alloy (σ = 3.2 MS/m) was prepared in a one-inch diameter tube. A wood cube (s = 1 cm) is immersed on the side of the tube (position = 10 relative in sensing region). This makes up a conductive body with inner void. Figure 16 shows spectral recostruction and its derivative. It is obvious in the image, there is low value centering around 40 kHz which indicates the void. This corresponds to a zero-valued line occurs between high-valued contours in the spectral derivative.

**Figure 17:**
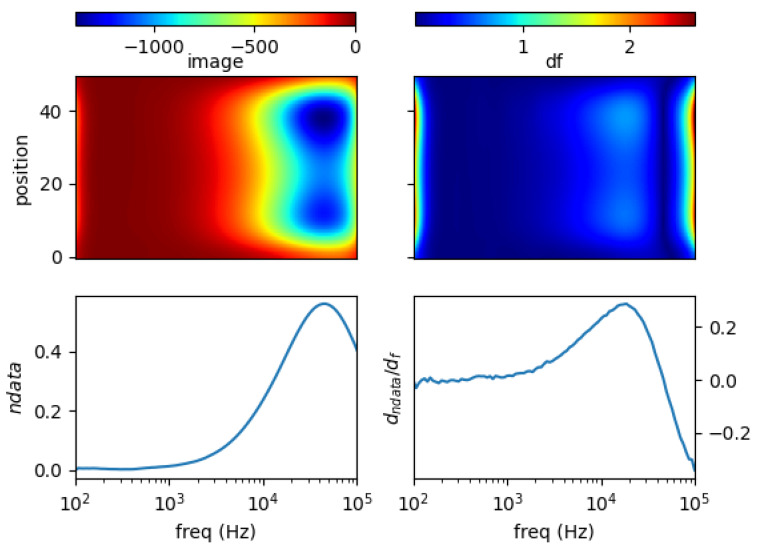
Spectral profile and its derivative (data and reconstructed image values) for plastic rods 0.25 inch at pos = 10, pos = 25, pos = 40 in liquid GaInSn 35 mL in a one-inch tube.

Three plastic rods (diameter = 0.25 inch) are inserted in the liquid metal tube, located at centre and both edges (position 10, 25 and 40) to construct multiple structural void in a conductive body. In Figure 17, spectral image reveals an elongated low-valued region from spatial positions 10–40. This should indicate the three inclusions, yet the sensitivity and regularisation effect fail to separate those voids. Accordingly, in the spectral derivative, there is a distinct zero-valued line at around 40 kHz cutting along the spatial position.

### 5.4. Spectral Derivative for Structural and Functional Classification

**Figure 18:**
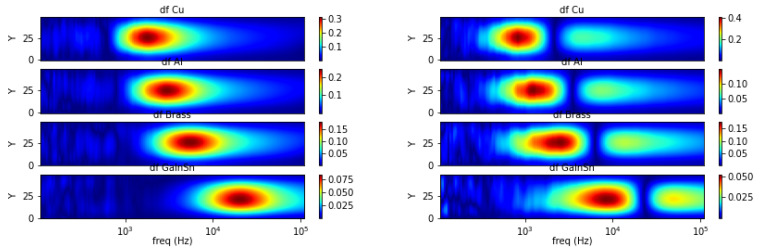
Z (**left**) and θ (**right**) spectral gradient (df) metallic samples with conductivity variations.

**Figure 19:**
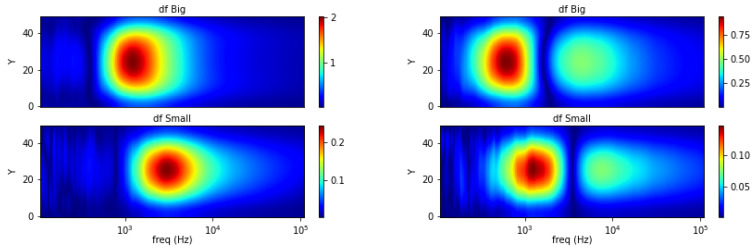
Z (**left**) and θ (**right**) spectral gradient (df) size variations.

**Figure 20:**
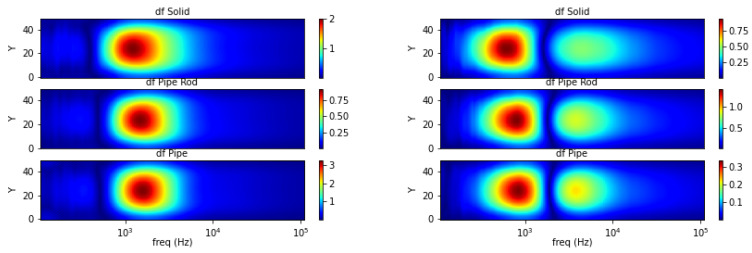
Z (**left**) and θ (**right**) spectral gradient (df) metallic structures.

Taking curve characteristics in Section 2, spectral gradient is applied to correlate metallic object circumstances with the respective image spectrums. The following Figure 18, Figure 19 and Figure 20 show spectral gradient of amplitude (Z) and phase (θ) reconstruction.

Figure 18 illustrates spectral derivative for amplitude and phase reconstructions from single sample with different conductivity levels (GaInSn, brass, aluminum and copper) at fixed position (*Y* = 25). Both amplitude and phase can be indications of different conductivity following particular location along the horizontal (frequency) axis. Peak values are the indicator on amplitude spectrum, whereas zero-valued lines across spatial (vertical) axis are the marker on phase spectrum.

Spectral gradient for aluminum rod with different diameter (small = 0.25 inch, large = 0.5 inch) at the centre region is plotted in Figure 19. While it is not straightforward from locus spectrum, the size variations can be inferred from the area of high-valued pixels. Larger area is associated with a larger sample and vice versa.

Three different structures are arranged from the same material (aluminum): pipe (hollow cylinder with outer diameter 0.5 inch and inner diameter 0.4 inch), pipe with 0.25 inch solid rod inside, and solid rod 0.5 inch. In the experiment, they are fixed at the centre (*Y* = 25) of sensing region. Figure 20 shows the respective gradient of amplitude and phase spectra. Aside from scrutinizing the value, it is difficult to distinguish the structure directly from spectral derivatives. Hence, the underlying Z-θ is further examined.

**Figure 21:**
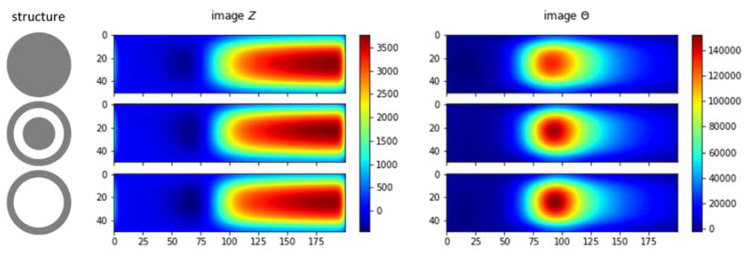
Z-θ spectrum metallic structures: hollow, hollow-with-inclusion, solid.

From only amplitude or phase spectrum as in Figure 21, it is also difficult to distinguish between structures. Therefore, normalised amplitude and phase images are combined (referring to method in Figure 10) to reveal both object conductivity (pronounce in amplitude image) and inner structure (phase signature).

**Figure 22:**
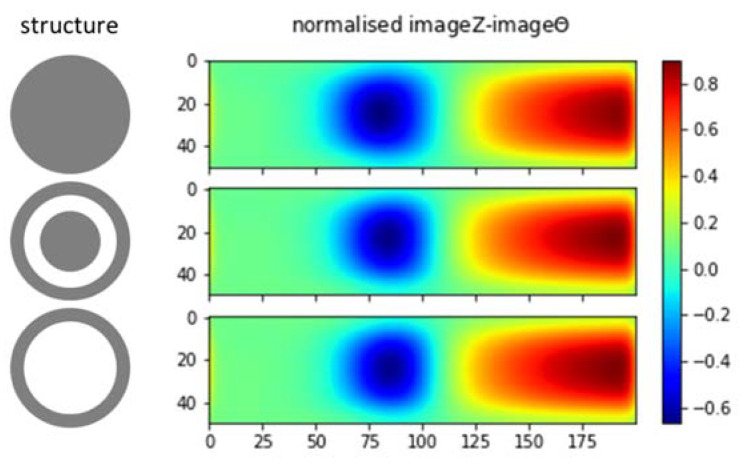
Combination of normalised Z-θ spectrum metallic structures: hollow, hollow-with-inclusion, solid.

Figure 22 shows subtraction of normalised phase spectrum from normalised amplitude spectrum. It can be seen that the negative spectral region is associated with the inner structure; while the positive value corresponds to the conductivity level. The interpretation of this spectral image fusion example can assist the subsequent post-processing to determine the object condition.

### 5.5. Complex Plot from Reconstruction

Analogous to Cole-Cole model [49] and dielectric spectroscopy [50], complex plots are also generated using reconstruction values to represent the behaviour. Region (group of pixels) making up the object is chosen for every investigated case where real and imaginary parts of the image are taken into account. Mean of pixel values in the region produces the following complex plots.

**Figure 23:**
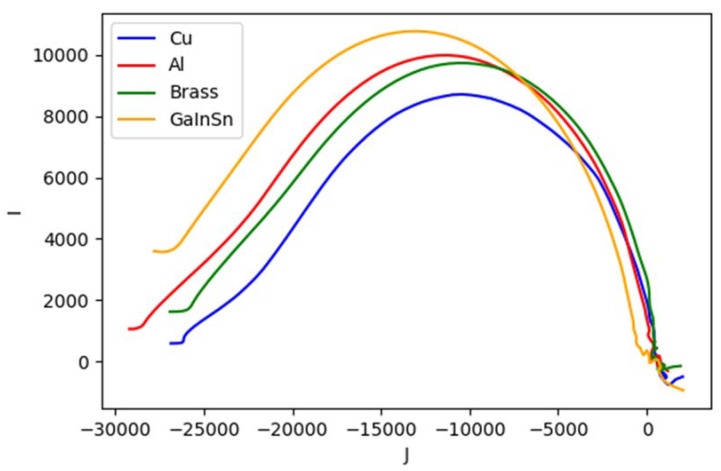
Complex plot of impedance for metallic samples with conductivity variations.

It can be seen from Figure 23 that metallic samples with different conductivity levels have similar shapes with different foci and vertices in the impedance plane. Here we allocate the horizontal axis J for imaginary value; the vertical axis I for real value. The plot starts from lower frequency near the origin (0,0) and curves to high frequency at the other end.

**Figure 24:**
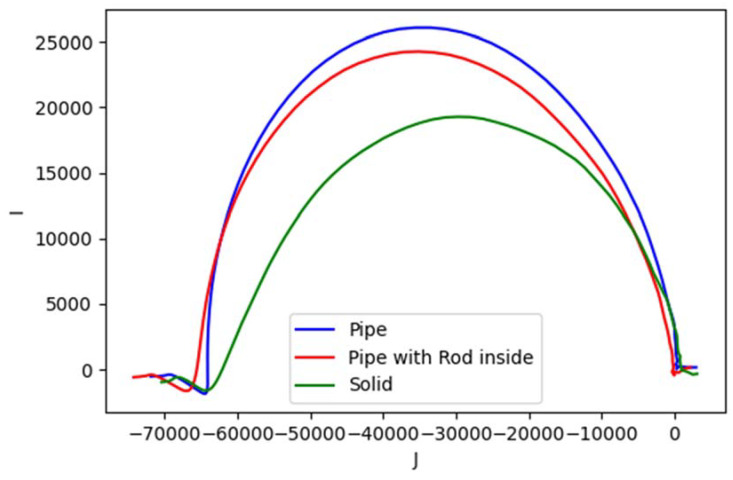
Complex plot of impedance for metallic structures.

Figure 24 represents structural circumstances of the aluminum body. The complex plots have the same shape with increasing vertices. The solid structure has the lowest vertex and the nearest focal point relative to the origin, while the pipe structure has the highest vertex. The vertex level of the pipe with the rod is between that of the solid and pipe, but it has the farthest focal point.

**Figure 25:**
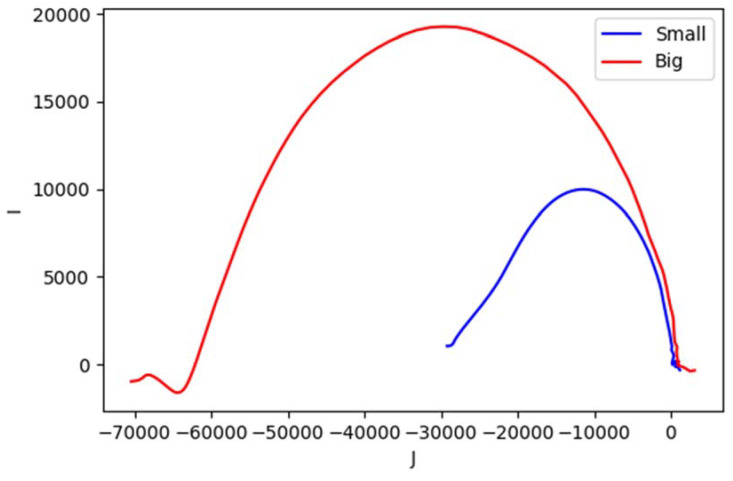
Complex plot of the impedance for size variations.

As for size variations (aluminum with diameter of: small = 0.25 inch, large = 0.5 inch), Figure 25 shows an obvious difference on the curves’ sizes.

**Figure 26:**
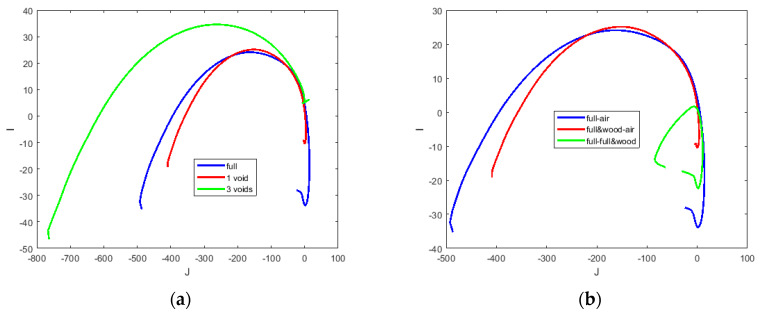
(**a**) Complex plot of the impedance for inclusions; (**b**) focusing on one void, but various background data.

The complex plot for the conductive liquid GaInSn with void distribution is depicted in Figure 26a. It is shown that although the curves’ shape are similar due to the embodiment of liquid metal, the level and inclination are varied for a single void and three distributed voids, respectively. This corresponds to the setup in Section 5.3. Focusing on 1 void a wooden cube inside of liquid metal, we can depict the cole-cole plot in three set of data, one pure metal from air background, one wood and liquid metal from air background, and finally liquid metal when the reference data is liquid metal including wooden block. Figure 26b shows various plots in these three situations.

**Figure 27:**
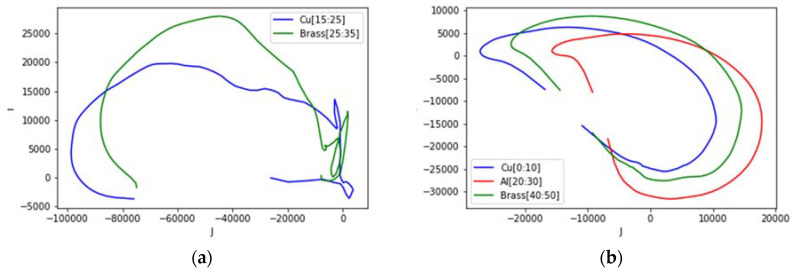
Complex plot of impedance for different samples at different locations: (**a**) two samples at left and right side; (**b**) three samples located at left, centre and right side.

The circumstances in Section 5.2 are plotted in Figure 27, taking the position of the sample in sensing region and evaluating their values in the confined area. For two different samples (a), the plot shows each curve that follows the trend in Figure 23 (copper and brass). However, for three samples (b), the curves are tilted to the side; still, each focal point and vertex are consistent with the trend for the samples with different conductivity levels.

## 6. Conclusions

This work offers an introduction to a spectrally-correlative MITS using both amplitude and phase data. Image reconstruction is plotted along a wide bandwidth with sufficient resolution, and its frequency derivative is exposed. Samples with conductivity, size, location and internal structure variations have been investigated. When a lower conductive sample, such as GaInSn, is mixed with a non-conductive sample the signature frequency is pushed slightly higher. Therefore, if lower conductive metals are of interest, the measurement must go to a higher frequency range to capture that effect. As opposed to a more conductive contrast (i.e., aluminum structure variations), the result shows that it is not the case as aluminum is much more conductive relative to the inner structure (air).

Although we used a TV (in fact, a spectral TV), which normally gives a sharp and clear image, in our iterative TV process we did not aim for a very sharp (near binary) image. There is a reason for this: if one obtains a very sharp boundary, one starts losing quantitative consistency with the measured data. Thus, the iterative TV is a good choice that gives a good balance between the image quantitative information and shapes. Image quantitative information is key in this study as it forms the basis of our complex Cole-Cole plots. Having said that, for future study more work needs to be conducted on hyperparameter tuning as when dealing with complex data and images in such a wide range of frequency. Additionally, for this wide frequency range, it is challenging to assign a parameter set that works very well in all these ranges. In our study, we had to maintain uniformity of these parameters so that we could produce reliable quantitative values. The same can be said for the measured data, as future studies will need to evaluate the noise performance for different frequencies, which may vary for real and imaginary parts.

The paper proposes the spatio-spectral method to characterize a metallic object in terms of electromagnetic and structural properties. The algorithm is explained and supporting experimental works are described. We have also presented complex plots from reconstruction which comprehensively indicate functional and structural behaviours in the metallic materials. This research is contributive in the context of eddy current, imaging, and induction spectroscopy of materials as significant information for characterization techniques.
